# Use of CD137 up-regulation to identify T cell receptors specifically reactive with mutated tumor associated antigens from tumor infiltrating lymphocytes

**DOI:** 10.1186/2051-1426-3-S2-P40

**Published:** 2015-11-04

**Authors:** Maria Parkhurst, Alena Gros, Pasetto Anna, Eric Tran, Jessica S Crystal, Todd Prickett, Paul Robbins, Steven A Rosenberg

**Affiliations:** 1 NIH/NCI/Surgery Branch, Bethesda, MD, USA; 2 NCI/NIH, Bethesda, MD, USA; 3 Surgery Branch/National Cancer Institute / National Institutes of Health, Bethesda, MD, USA; 4 NCI/NIH, Rutgers Robert Wood Johnson Medical School, New Brunswick, NJ, USA; 5 NIH/NCI, Bethesda, MD, USA

## Background

The adoptive transfer of tumor infiltrating lymphocytes (TIL) can mediate the regression of metastatic melanoma. In addition, the adoptive transfer of lymphocytes genetically modified to express tumor reactive T cell receptors (TCRs) can mediate tumor regression. Many T cells from TIL recognize mutated antigens expressed only on the autologous patient's tumors. Therefore, we attempted to isolate TCRs reactive with unique mutated antigens so that we might eventually be able to treat patients with autologous T cells genetically modified to express those TCRs.

## Methods

Mutations in tumors were identified using whole exome sequencing and/or RNA sequencing. Tandem minigene (TMG) constructs containing 12-24 minigenes were synthesized, each encoding the mutated amino acid flanked by 12 amino acids on both sides. RNAs encoding the TMGs were *in vitro* transcribed and electroporated into autologous dendritic cells (DCs). Recognition of TMGs by TIL was evaluated on the basis of IFN-γ secretion and CD137 expression after overnight coculture with the electroporated DCs. Subsequently, mutation reactive T cells were enriched from TIL by sorting for CD137+ T cells after overnight coculture with the electroporated DCs and were expanded *in vitro* with anti-CD3 and IL-2. Dominant TCR α and β chain sequences were identified in the enriched mutation reactive populations, and retroviruses encoding those TCRs were used to transduce human PBL to determine if they mediated recognition of the mutated antigen.

## Results

Thus far, using these techniques we have isolated mutation reactive TCRs from 6 different patients with metastatic melanoma as described in the attached table. We are currently extending these techniques to identify mutation reactive TCRs for patients with other cancers including those of the gastrointestinal tract, breast, and ovaries. We are also developing clinical reagents to treat patients with TCRs that recognize unique mutations on autologous tumor cells.

**Table 1:** Mutation reactive TCRs identified by CD137 upregulation.

Patient	Mutated antigen	# of independent TCRs
3466	COL18A1	1
3466	ERBB2	1
3903	KIAA1279	3
3903	KIAA1967	1
3903	PHKA1	1
3784	FLNA	1
3784	KIF16B	3
3678	FBOX21	1
3678	RECQL5	2
3716	Not yet identified at individual gene level	1
4000	Not yet identified at individual gene level	Up to 4

